# Gentle rocking movements during sleep in the elderly

**DOI:** 10.1111/jsr.12989

**Published:** 2020-02-15

**Authors:** Rachel van Sluijs, Elisabeth Wilhelm, Quincy Rondei, Ximena Omlin, Francesco Crivelli, Dominik Straumann, Lukas Jäger, Robert Riener, Peter Achermann

**Affiliations:** ^1^ Department of Health Science and Technology Sensory‐Motor Systems Laboratory Institute of Robotic and Intelligent Systems Swiss Federal Institute of Technology Zurich Switzerland; ^2^ Sleep & Health Zurich University Center of Competence University of Zurich Zurich Switzerland; ^3^ Nuffield Department of Clinical Neurosciences Sleep and Circadian Neuroscience Institute University of Oxford Oxford UK; ^4^ Medical Faculty University of Zurich Zurich Switzerland; ^5^ Department of Psychiatry, Psychotherapy and Psychosomatics The KEY Institute for Brain Mind Research University Hospital of Psychiatry Zurich Switzerland; ^6^ Institute of Pharmacology and Toxicology University of Zurich Zurich Switzerland

**Keywords:** mother movement, movement intervention, otolith, sensory mismatch, sleep laboratory

## Abstract

Vestibular stimulation in the form of rocking movements could be a promising non‐pharmacological intervention for populations with reduced sleep quality, such as the elderly. We hypothesized that rocking movements influence sleep by promoting comfort. We assessed whether gentle rocking movements can facilitate the transition from wake to sleep, increase sleep spindle density and promote deep sleep in elderly people. We assessed self‐reported comfort using a pilot protocol including translational movements and movements along a pendulum trajectory with peak linear accelerations between 0.10 and 0.20 m/s^2^. We provided whole‐night stimulation using the settings rated most comfortable during the pilot study (movements along a pendulum trajectory with peak linear acceleration of 0.15 m/s^2^). Sleep measures (polysomnography) of two baseline and two movement nights were compared. In our sample (*n* = 19; eight female; mean age: 66.7 years, standard deviation: 3 years), vestibular stimulation using preferred stimulation settings did not improve sleep. A reduction of delta power was observed, suggesting reduced sleep depth during rocking movements. Sleep fragmentation was similar in both conditions. We did not observe a sleep‐promoting effect using settings optimized to be comfortable. This finding could imply that comfort is not the underlying mechanism. At frequencies below 0.3 Hz, the otoliths cannot distinguish tilt from translation. Translational movement trajectories, such as used in previous studies reporting positive effects of rocking, could have caused sensory confusion due to a mismatch between vestibular and other sensory information. We propose that this sensory confusion might be essential to the sleep‐promoting effect of rocking movements described in other studies.

## 1 INTRODUCTION

Sleep changes with age (Carrier & Bliwise, 2003). Elderly people experience a more fragmented sleep with more awakenings (Cooke & Ancoli‐Israel, 2010) and a rise in the percentage of time spent in lighter sleep stages (stages 1 and 2; Carrier, 2010). The prevalence of chronic sleep disturbances in elderly people ranges from 40% to 70% (Van Someren, 2000). About 20% complain about early morning awakenings and about 50% of older subjects report difficulties initiating or maintaining sleep (Crowley, 2011; Welsh & Ptacek, 2010). These changes in sleep have consequences for general physical and mental health, as well as for autonomy and self‐care (Van Someren, 2000). An increased risk of falls, difficulty with concentration and memory, and overall decreased quality of life are results of insufficient sleep quality in the elderly (Ancoli‐Israel, Ayalon, & Salzman, 2008).

To manage such sleep complaints, 10% – 27% of elderly people use sleep medication on a daily basis (Krystal, 2010; Van Someren, 2000). Pharmacological therapies come with a high risk of dependency and often lead to sedation the following day, increasing the risk of falls and hip fractures in elderly people (Donnelly, Bracchi, Hewitt, Routledge, & Carter, 2017). Therefore, non‐pharmacological treatment approaches, such as improving sleep hygiene, cognitive behavioural therapy and relaxation therapies, are increasingly recognized (Gehrman & Gooneratene, 2010). Recently, vestibular stimulation in the form of gentle rocking movements has been proposed as a promising therapeutic modality. Sleep‐promoting effects reported in young adults include facilitation of wake‐to‐sleep transition (Bayer et al., 2011; Perrault et al., 2019; Woodward, Tauber, Spielmann, & Thorpy, 1990) and more deep sleep (Perrault et al., 2019; Shibagaki, Ashida, Morita, Ikeura, & Yokoyama, 2017).

The aim of this study is to assess whether gentle rocking movements can promote sleep in older adults (>60 years of age). We hypothesized that rocking movements influence sleep by promoting comfort. To find out if one choice of settings was experienced as more comfortable and sleep inducing we provided vestibular stimulation along a translational or pendulum trajectory (head‐to‐toe and side‐to‐side direction) with different intensities (linear acceleration between 0.10 and 0.20 m/s^2^; rotational accelerations between 1.43 and 2.87 °/s^2^) in a pilot protocol. We used the stimulation settings rated as most comfortable and most sleep inducing for a night‐time sleep study. We hypothesized that gentle rocking movements would shorten time to sleep onset, promote deep sleep and increase the number of sleep spindles, with positive effects on memory function the next morning.

## 2 METHODS

### 2.1 Participants

The data of 19 right‐handed participants (eight female; mean (M) age 66.7 years, standard deviation (*SD*) 3 years) were recorded and analysed. Only non‐smokers between 60 and 75 years of age, with a body mass index between 19 and 30, and with moderate caffeine/alcohol consumption (<5 consumptions containing caffeine/day, <10 alcoholic drinks/week) were included. Participants with a diagnosed sleep disorder or signs of a sleep disorder in the laboratory (sleep apnea, myoclonus and insomnia), use of medication known to influence sleep, engaging in shift work, who recently travelled across time zones, slept <6.5 hr or >7.5 hr on an average night or habitually napped for longer than 1 hr a day were excluded. Eligibility was assessed using questionnaires (Bloch, Schoch, Zhang, & Russi, 1999; Johns, 1991) and polysomnography (PSG) during a screening night.

The Ethical Committee of the Canton Zürich (KEK‐ZH‐Nr. 2017–00093) and the Swiss Agency for Therapeutic Products (Swissmedic 10000145) approved the study protocol. The trial was registered at clinicaltrials.gov (NCT03133442). Due to setup constraints, no participants over 1.90 m or weighing more than 110 kg were included (Crivelli, Heinicke, Omlin, & Riener, 2014).

### 2.2 Stimulation settings

Stimulation was provided using two previously described automated beds, the Somnomat A and B (Crivelli et al., 2014). In summary, these devices are composed of a one‐person bed mounted on a moving platform. The Somnomat A provides translational movements from head to toe or from side to side. The Somnomat B moves in two directions along a pendulum trajectory with a rotational centre 4 m above the bed. Gentle rocking movements were provided in the form of harmonic oscillations.

**Figure 1:**
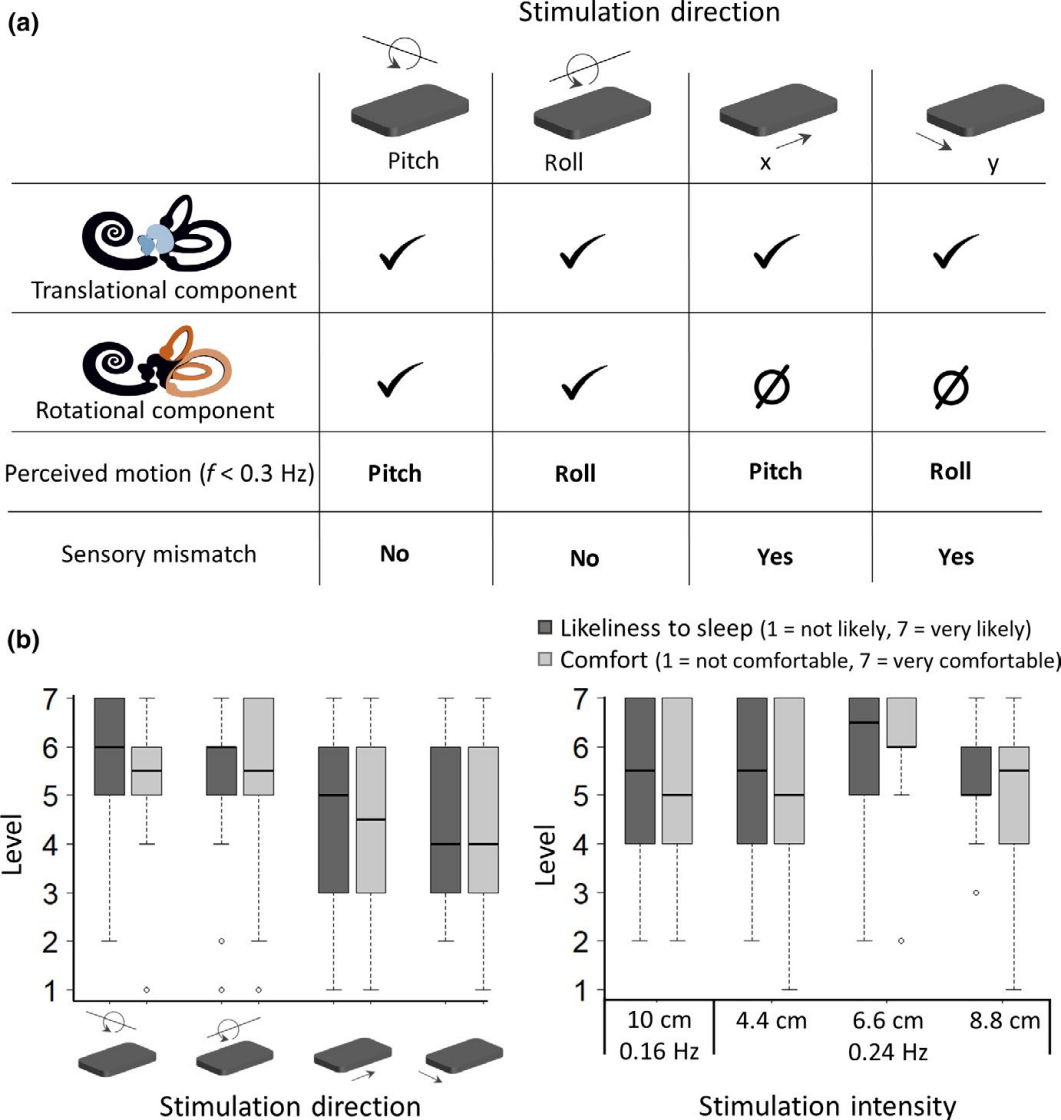
Selection of stimulation settings. (a) Stimulation of the different components of the inner ear depending on stimulation direction. The otoliths cannot distinguish translation from angular motion and at frequencies below 0.3 Hz this leads to the subjective perception of angular motion. The mismatch between the vestibular sensation of rotation and the translational movement perceived by other senses might lead to sensory mismatch. Saccule and utricle (blue), semicircular canals (orange). (b) Subjective experience of four movement directions and four amplitude frequency combinations of healthy elderly people (*n* = 14) who participated in our pilot study. Likert items for ‘likeliness to fall asleep’ and comfort are on a Likert item scale with 1 = not likely to fall asleep/not comfortable and 7 = very likely to fall asleep/very comfortable. Dots are outliers

We determined the settings perceived as most comfortable and sleep promoting by our target population in a pilot protocol. The data of 14 participants (eight female; aged (M) 65.0 years, (*SD*) 3 years) without disorders of the vestibular system or sensitivity to motion sickness (Motion Sickness Susceptibility Questionnaire <10 (Golding, 2006)) were collected. Comfort and likeliness of falling asleep were rated on a scale from 1 to 7, with 1 = not likely to fall asleep/not comfortable and 7 = very likely to fall asleep/very comfortable. Participants tried out four different movement directions (at randomly assigned linear acceleration between 0.10 and 0.20 m/s^2^) while lying in the bed in their self‐reported habitual sleeping position: head‐to‐toe along a pendulum trajectory (pitch), side‐to‐side along a pendulum trajectory (roll), head‐to‐toe lateral motion (x) and side‐to‐side lateral motion (y; Figure 1a). Their favourite stimulation direction was used to present four amplitude‐frequency combinations (I: A = 0.10 m, f = 0.16 Hz; II: A = 0.044 m, f = 0.24 Hz; III: A = 0.066 m, f = 0.24 Hz; IV: A = 0.088 m, f = 0.24 Hz). The amplitude‐frequency combination resulted in peak linear accelerations perceived by the otoliths between 0.10 and 0.20 m/s^2^, with a rotational component perceived by the semicircular canals between 1.43 and 2.87°/s^2^. Both linear and rotational accelerations are above the reported perception threshold (Clark & Stewart, 1970; Walsh, 1961).

The difference in comfort and sleepiness between the four directions was not significant (Figure 1b, repeated measures ANOVA, *p* > .05). However, the rotational movements were on average perceived as more comfortable than the two translational movements. Therefore, the participants in the sleep study could select the direction of stimulation (roll or pitch) prior to the first experimental night. Also, the rating of the different stimulation intensities did not significantly differ, but a trend towards a preference for movements with a frequency of 0.24 Hz and an amplitude of 0.066 m was observed (repeated measures ANOVA square transformed comfort: *F*(3,39) = 2.776, *p* = .054). Therefore, this amplitude and frequency, resulting in peak rotational accelerations of 2.15 °/s^2^ and peak linear accelerations of 0.15 m/s^2^, was used for all participants in the sleep study.

### 2.3 Study protocol

Participants slept for four nights in the Somnomat: two consecutive 7‐hr nights in the stationary bed with the playback of a sound recording of the moving bed (baseline nights; BN) and two consecutive 7‐hr nights with the bed moving from lights off to lights on (movement nights; MN). Block order (BN or MN) was randomized. Measurements were scheduled on the same weekdays, with 5 days in between for all participants except one for whom measurements were separated by 3 weeks. Participants were not restricted in their movement and did not receive instructions regarding the sleeping position.

As a preparation for the nights in the laboratory, participants kept a 7‐hr regular sleep‐wake cycle at their self‐chosen bedtime for 7 days. Additionally, participants abstained from caffeine and alcohol consumption for the last 3 days prior to the laboratory visit. Compliance was checked using a sleep diary, actigraphy and alcohol breath tests.

### 2.4 Polysomnography

Polysomnography was measured during the entire 7‐hr period from lights off to lights on. Electroencephalogram (EEG) electrodes were applied according to the 10–20 system (F3, F4, C3, C4, P3, P4, O1, O2, A1, A2) and referenced to Cz. Signals were amplified (Micromed), filtered with a high‐pass filter (EEG: −3 dB at 0.15 Hz; EMG: 10 Hz; ECG: 1 Hz) and an anti‐aliasing low‐pass filter (−3 dB at 67.4 Hz), sampled at 256 Hz and recorded (Rembrandt DataLab Version 8.0; Embla Systems).

For analysis, the EEG signals were re‐referenced to the contralateral mastoids (A1, A2). Each 20‐s epoch was visually attributed to a sleep stage according to standard criteria (Iber, Ancoli‐Israel, Chesson, & Quan, 2007). Artefacts were marked during visual inspection of the data.

Latencies to stages N1, N2 and N3 were defined as the time from lights off until the occurrence of two consecutive epochs of the respective sleep stage. The latency to N2 will be referred to as sleep onset (SO). Furthermore, the duration of the first episode of stage N1 (defined by some as sleep latency (Bayer et al., 2011)) was calculated as the difference between the first occurrence of N1 and the first occurrence of N2, and the duration of initial stage N2 was calculated as the difference between the first occurrence of stage N2 and the first occurrence of N3. Time in bed (TIB) was defined as the time from lights off until lights on the next morning. Total sleep time (TST) was defined as TIB minus time awake. As measures for sleep fragmentation, we calculated the number of sleep stage shifts per hour, the number of shifts to lighter sleep stages (from rapid eye movement (REM) sleep to wake and from non‐REM stages to a lighter non‐REM stage or wake) and the number of night‐time awakenings (shifts to wake). Sleep efficiency was calculated as % of TST of TIB. To check that the motor of the bed did not create additional artefacts in the signal, we compared the number of artefacts for nights with and without movement.

For spectral analysis of the EEG, the signal was transformed using a Fast Fourier Transform (Hanning window; averages over five 4‐s epochs). Average non‐REM sleep EEG spectra were calculated using the common number of non‐REM sleep epochs across the four recordings of a participant and power in different frequency bands was compared. We also analysed the build‐up of delta power (0.75–4.5 Hz) during the first 20 min after sleep onset (time defined by the maximum common length before the first wake episode). The slope of the build‐up of delta activity was calculated as delta activity 20 min after SO minus delta activity at SO divided by 20 min.

Detection of slow oscillations and sleep spindles was performed using previously described algorithms (Bersagliere & Achermann, 2010; Ferrarelli et al., 2007). The signal of the C4‐A1 derivation was down‐sampled to 128 Hz and band‐pass filtered in forward and backward directions for both the slow oscillation analysis (third‐order Chebyshev type II high‐pass filter with a cut‐off frequency of 0.4 Hz; sixth‐order Chebyshev type II low‐pass filter with a cut‐off frequency of 2.4 Hz) and the spindle analysis (sixth‐order Chebyshev type II band‐pass filter; −3 dB at 12 Hz; −3 dB at 15 Hz). A slow oscillation was detected when the signal between two consecutive zero‐crossings surpassed an amplitude threshold set at 25 μV (corresponding to 37.5 μV in the unfiltered signal (Iber et al., 2007)). The average frequency, duration and maximum amplitude of the slow waves was determined (Bersagliere & Achermann, 2010). The density of slow waves was calculated as the number of slow waves per 20‐s epoch. A spindle was detected whenever the signal amplitude exceeded six times the average amplitude (upper threshold) and the duration of the spindle had to be 0.5–3 s (Iber et al., 2007). The average frequency, duration, maximum amplitude, integrated absolute amplitude and activity (integrated absolute amplitude/min) of each spindle were determined (Ferrarelli et al., 2007). Spindle density was calculated as number of spindles per 20‐s epoch.

### 2.5 Memory performance

Declarative memory performance was assessed using a previously described word‐pair task (Plihal & Born, 1997; Figure S1). In summary, participants learned a list of 40 semantically related pairs of German nouns 1 hr before lights off, followed immediately by a recall test of all words. During the immediate recall (IR) subjects received feedback and were presented with the correct word pairs. Delayed recall (DR) took place half an hour after lights on. A different list of word pairs was used for each experimental night and their order was randomized.

One point was awarded for correctly remembered pairs and half a point for pairs containing spelling or single‐plural mistakes. Overnight performance improvement was calculated as the difference between immediate and delayed recall. The initial acquisition rate was the immediate recall score expressed as a percentage of the delayed recall score (Lustenberger, Maric, Dürr, Achermann, & Huber, 2012).

### 2.6 Statistics

Statistical analysis was performed using R Studio version 1.2.1335, using the linear mixed‐effects model package lme4 (Bates, Mächler, Bolker, & Walker, 2015). For each outcome measure, a linear mixed model was made. The experimental condition (baseline vs. movement), night (first or second consecutive night) and the interaction between condition and night were the fixed effects and participant (repeated measures) a random effect:

outcome variable ~ condition*night + (1|participant) + ε

The level of significance was set at *p* = .05.

## 3 RESULTS

### 3.1 Movement selection

After trying out both movement directions, 10 participants chose the roll movement and nine participants the pitch movement. Both directions were rated as equally comfortable (Roll: M, 5.8; *SD*, 1.3; pitch: M, 5.8; *SD*, 1.5; with 7 being very comfortable) and similarly sleep inducing (Roll: M, 5.8; *SD*, 1.4; pitch: M, 5.4; *SD*, 1.4; with 7 giving a very high chance of falling asleep). The preference for their chosen direction was rated 1 point more comfortable (*SD*, 1.1) and 0.7 points more sleep inducing (*SD*, 0.7) than the other direction.

### 3.2 Effect on sleep

**Figure 2:**
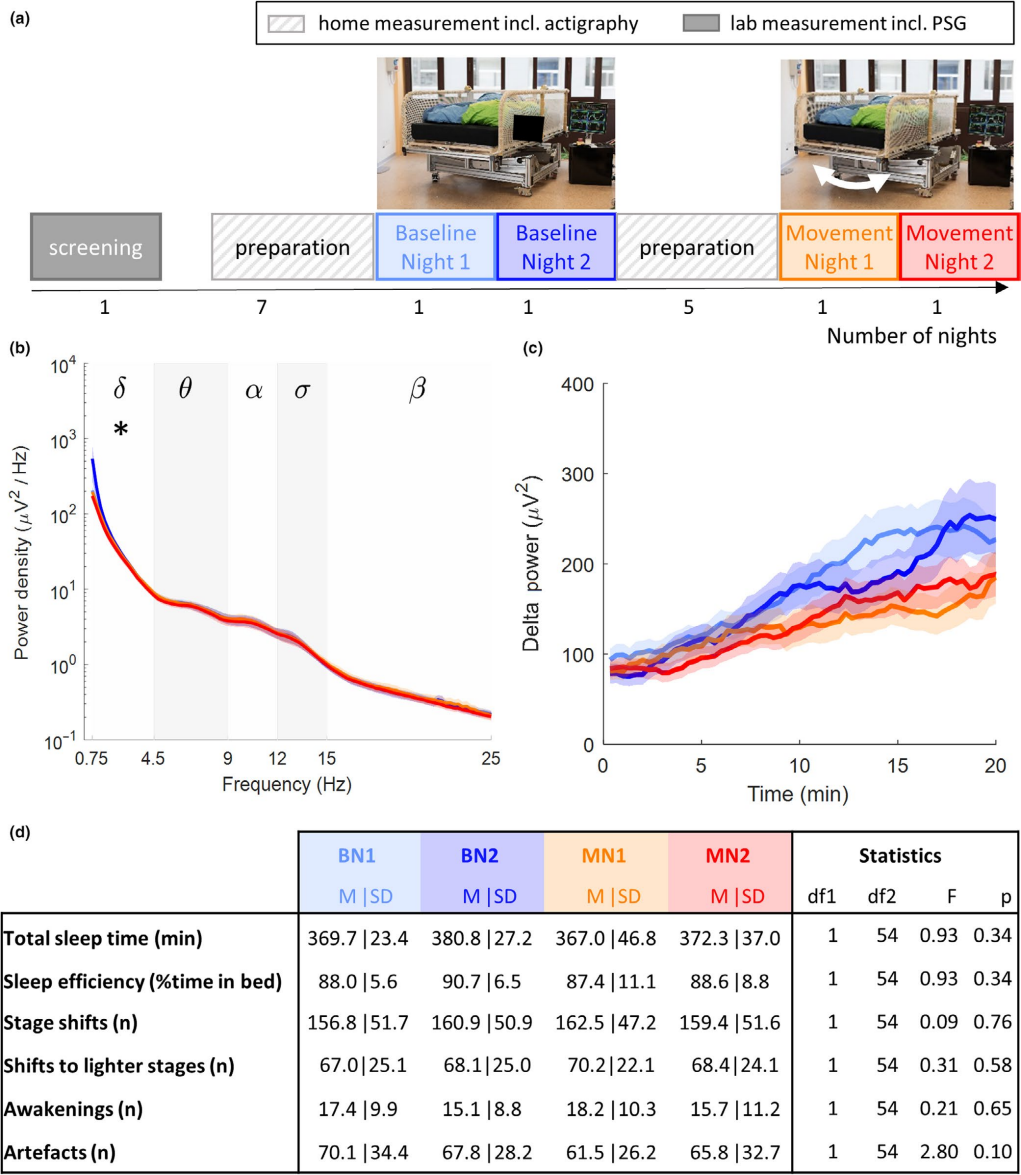
Protocol and results of the sleep study. (a) Study protocol. (b) Average power‐density spectra of EEG derivation C4‐A1 during stage N3. Analysed frequency bands: delta (δ, 0.75–4.5 Hz), theta (θ, 4.5–9 Hz), alpha (α, 9–12 Hz), sigma (σ, 12–15 Hz) and beta (β, 15–25 Hz). Lines are mean power density, shading is *SEM*. The star represents the significant difference between conditions (linear mixed‐effects model for condition (*p* < .05)). (c) Build‐up of delta power (0.75–4.5 Hz) during the first 20 min after sleep onset. Lines represent mean power and the shading the *SEM*. For mean power and *SD* per frequency band see Table S2. (d) Sleep architecture variables are based on the visual scoring of 20‐s epochs according to standard criteria (Iber et al., 2007). Statistics relate to the fit of a linear mixed model with condition as fixed factor and participant and night (first or second consecutive night) as random factors. *p*‐values refer to condition. BN, baseline night; EEG, electroencephalogram; M, mean; MN, movement night; PSG, polysomnography; *SD*, standard deviation; *SEM*, standard error of the mean

Sleep architecture, including sleep efficiency (Figure 2c), latency to different sleep stages and time spent in these sleep stages, did not significantly differ between the movement and baseline nights (Table S1). Even though no changes in sleep architecture were observed, spectral analysis of the sleep EEG revealed a decrease of delta power (0.75–4.5 Hz) from 232.0 µV^2^ (*SD*, 113.1 µV^2^) during the second baseline night to 187.5 µV^2^ (*SD*, 77.3 µV^2^) during the second movement night (*p* = .03; Figure 2b). The build‐up of power in this frequency band during the first 20 min after sleep onset seems to be steeper in nights without stimulation (M_BN2_: 10.7 µV^2^/min; *SD*_BN2_: 16.5 µV^2^/min) compared to nights with stimulation (M_MN2_: 6.3 µV^2^/min; *SD*_MN2_: 8.4 µV^2^/min), although this effect was not significant (Figure 2c). No changes in other frequency bands were observed (Table S2). Also, the number and density of slow oscillations did not significantly differ between movement and baseline nights (Table S3).

To investigate whether the reduced sleep intensity in the movement condition affected sleep fragmentation, we calculated the number of sleep‐stage shifts, the number of shifts to lighter sleep stages and the number of night‐time awakenings. No significant changes in these variables were observed (Figure 2d). The number of artefacts (including movement artefacts and arousals) was not significantly influenced by the movement of the bed (Figure 2d).

Lastly, the number and density of sleep spindles did not significantly differ between movement and baseline nights (Table S4). A trend towards a reduction of the integrated amplitude of the spindles (ISA) during nights with stimulation was observed compared to nights without stimulation (Table S4; *p* = .05). We observed a significant improvement from the first to the second consecutive night in both immediate and delayed recall scores (Figure S1 and Table S4). The higher immediate recall scores during the second night was independent of the experimental condition. A significant interaction between experimental night and condition was observed regarding the delayed recall score, with participants performing less well on the morning after the first night with motion (M_MN1_: 14.2; *SD*_MN1_: 6.5) compared to baseline (M_BN1_: 17.9; *SD*_BN1_: 7.7), but better on the morning after the second night with motion (M_MN2_: 19.0; *SD*_BN2_: 9.0) compared to baseline (M_BN2_: 17.2; *SD*_BN2_: 7.8) (*p* < .05). These changes were not reflected in the overnight improvement of the memory task performance.

## 4 DISCUSSION

Vestibular stimulation using subjectively preferred stimulation direction and intensity did not improve sleep in our sample of elderly participants. Unlike studies in younger adults, we did not observe a facilitation of sleep onset, more deep sleep or an increase in the number or density of sleep spindles (Omlin et al., 2018; Perrault et al., 2019; Shibagaki et al., 2017; Woodward et al., 1990). On the contrary, we observed reduced delta power, suggesting a reduced sleep depth under the influence of rocking movements. Furthermore, a trend towards a reduction of the integrated amplitude of the spindles during nights with stimulation was observed. However, this did not seem to influence memory performance, because no effect of motion on overnight improvement was observed. The reported improvement in immediate and delayed recall scores from the first to the second consecutive night could be a first night or learning effect. It is important to mention that we did not observe a change in the number of sleep‐stage changes, the sleep fragmentation index or the number of artefacts, indicating that sleep fragmentation was similar in both conditions.

Participants were not restricted in their movement and did not receive instructions regarding sleeping position. Because the degree of otolithic stimulation depends on the head orientation with respect to the applied motion (Eron, Cohen, Raphan, & Yakushin, 2008; Jaeger, Takagi, & Haslwanter, 2002; Walsh, 1961), the vestibular stimulation received varied from person to person and throughout the night. Regardless of this, the otoliths (perception of linear accelerations) were stimulated in all stimulation setting and sleeping positions (Figure 1a). A study in rodents showed that these linear accelerations were important for the promotion of sleep (Kompotis et al., 2019). Like most sensory organs, the vestibular system's anatomy (Rauch, Velazquez‐Villasenor, Dimitri, & Merchant, 2001) and function (Baloh, Enrietto, Jacobson, & Lin, 2001) change with age. Thus, it might be that the linear peak acceleration of 0.15 m/s^2^ used in this study, although above the average perception threshold in adults aged 18–40 years (0.064 m/s^2^), was too low (i.e., too close to the perception threshold) to promote sleep in adults over 60 years of age. Indeed, we are at the lower limit of the intensities (accelerations) that have been tested in other studies (Bayer et al., 2011; Omlin et al., 2018; Perrault et al., 2019; Shibagaki et al., 2017; Woodward et al., 1990). In a recent study on the effect of rocking movements with different stimulation intensities (0.15–0.35 m/s^2^) on nap sleep there was no indication of an intensity‐dependent effect. The data suggest that also rocking movements with linear peak acceleration of 0.15 m/s^2^ influence sleep in young adults (van Sluijs et al., 2020).

We previously hypothesized that rocking movements influence sleep by promoting comfort, inducing sleepiness and promoting relaxation (Crivelli et al., 2014; Omlin et al., 2018). Therefore, we chose our stimulation settings to be perceived as optimally comfortable and sleepiness inducing by the target population of our intervention. However, these subjectively preferred settings did not positively influence sleep, suggesting promotion of relaxation through comfort might not be the mechanism underlying the sleep‐promoting effect of rocking. For their study in young adults, Bayer and colleagues selected a set of motion parameters that generated stimulation while minimizing physical discomfort (Bayer et al., 2011). Potentially, stimulation with a higher intensity that reduced comfort, but did not create discomfort (comfort rating below neutral), could be a valid strategy.

Another proposed mechanism is the synchronization of brain waves facilitated by the direct anatomical connections between the vestibular sensory cortex and sleep centres (Bayer et al., 2011). Indeed, one study reported an entrainment of slow waves to the movement of a rocking bed moving with a frequency of 0.25 Hz (Perrault et al., 2019). We chose a frequency (0.24 Hz) that allows entrainment of slow waves (0.4–4.5 Hz). However, using this frequency was not sufficient in our sample to improve sleep, suggesting that besides frequency other stimulation parameters play a role in intervention success.

An alternative explanation for the difference between our findings and previous studies in rodents and humans could be related to the rotational component of the stimulation we provided. At frequencies below 0.3 Hz the otoliths cannot distinguish tilt from translation. During pure translational stimulation a sensory mismatch arises between vestibular information and other streams of sensory information (Reason, 1970). This sensory conflict is the underlying cause of motion sickness, which is often accompanied by tiredness. The gentle movements provided in studies investigating the effect of rocking movements on sleep (Bayer et al., 2011; Omlin et al., 2018; Perrault et al., 2019; Shibagaki et al., 2017; Woodward et al., 1990) are too subtle to induce motion sickness but might evoke tiredness through this mismatch. When a rotational component is added to the stimulation, it is clear to the body that the stimulation is tilt and, thus, the sensory mismatch is solved. This could explain the difference between our findings and previous reports of a promotion of sleep through rocking movements (Bayer et al., 2011; Perrault et al., 2019; Shibagaki et al., 2017). Twelve out of 18 subjects in the study by Omlin and colleagues selected movements with a rotational component, and this could explain why also in this study only a weak effect of vestibular stimulation on sleep was observed (Omlin et al., 2018).

Sensory mismatch has been reported as the main cause of the Sopite syndrome (Walton, Lamb, & Kwok, 2011). Sopite syndrome is a set of symptoms, including drowsiness, lethargy and reduced attention (Graybiel et al., 1965; Lawson & Mead, 1998), which may occur during exposure for extended periods of time to gentle low‐frequency motion. Changes in brain activity related to Sopite syndrome include a slowing of brain waves during wake (Wood et al., 1990), higher total sleep time (Graybiel et al., 1965) and sudden/accelerated sleep onset (Lawson & Mead, 1998). This is in line with the slower theta activity during wake and longer total sleep time reported by Kompotis and colleagues (Kompotis et al., 2019) and the shorter sleep onset latency reported by Woodward and colleagues (Woodward et al., 1990) and Perrault and colleagues (Perrault et al., 2019). Also in our study, we observe an influence of motion on the frequency composition of brain activity around sleep onset. If our sensory‐mismatch hypothesis is true, this influences our understanding of the neural circuitry responsible for the sleep‐promoting effects of vestibular stimulation. Moreover, inhibition of noradrenergic neurons in the locus coeruleus involved in sensory information processing is thought to contribute to drowsiness (Nishiike, Kubo, Nakamura, & Suetaka, 2001) and possibly promotion of sleep.

In a next step, a systematic comparison between movements with and without a rotational component regarding the degree to which they cause sensory mismatch, and concerning their effectiveness in promoting sleep, is needed. Because sleeping problems are prominent in older adults, this population can greatly benefit from a non‐pharmacological therapeutic modality. A better understanding of the underlying mechanism is needed to design an effective rocking‐based therapy for this target population.

## CONFLICT OF INTEREST

No conflicts of interest declared.

## AUTHOR CONTRIBUTION

The study was designed by XO, RR, PA, DS and RS. Data were collected and post‐processed by FC, RS, QR and LO. Data analysis was performed by DS, XO, LJ, RS and PA. The manuscript was written by RS, DS, PA and EW. The manuscript was critically reviewed by all co‐authors.
