# Supplementary material for: Gentle rocking movements during sleep in the elderly
Source: J Sleep Res. 2020 Feb 15;29(6):e12989. doi: 10.1111/jsr.12989 (PMC7757236; doi:10.1111/jsr.12989)
Supplement: Supplementary file 1 [file JSR-29-e12989-s001.docx]

**Supplementary material**

GENTLE ROCKING MOVEMENTS DURING SLEEP IN THE ELDERLY

Rachel van Sluijs^1,2^, Elisabeth Wilhelm^1,2^, Quincy Rondei^1^, Ximena Omlin^1,3^, Francesco Crivelli^1^, Dominik Straumann^4^, Lukas Jäger^1^, Robert Riener^1,2,4†^ & Peter Achermann^2,5,6†^

^1^Sensory-Motor Systems Lab, Institute of Robotic and Intelligent Systems, Department of Health Science and Technology, Swiss Federal Institute of Technology, Zurich, Switzerland

^2^Sleep & Health Zurich, University Center of Competence, University of Zurich, Zurich, Switzerland

^3^Sleep and Circadian Neuroscience Institute, Nuffield Department of Clinical Neurosciences, University of Oxford, UK

^4^Medical Faculty, University of Zurich, Zurich, Switzerland

^5^The KEY Institute for Brain Mind Research, Department of Psychiatry, Psychotherapy and Psychosomatics, University Hospital of Psychiatry, Zurich, Switzerland

^6^Institute of Pharmacology and Toxicology, University of Zurich, Zurich, Switzerland3

^†^Shared last authorship

***Correspondence:**Rachel van Sluijs, ETH Zurich, Sonneggstrasse 3, CH-8092 Zurich, Switzerland, +41 632 07 14, rachel.vansluijs@hest.ethz.ch

**Table S1. Sleep Architecture.** Consecutive 20-s epochs of the recordings were visually scored according to the AASM criteria (Iber, Ancoli-Israel, Chesson, & Quan, 2007) by a blinded scorer. Latencies and durations were derived from the visual scoring. Artefacts were marked during visual inspection of the data. A linear mixed model with condition, night (first or second consecutive night) and the interaction between condition and night as fixed factors and participant as random factor was used. Df1, df2, F and p of the main effect of Condition are given in the table. Bold numbers indicate significant effects of condition (p < 0.05). Star indicates significant main effect of night (p < 0.05). No significant interaction effects were present.

|  | **BN1** | | **BN2** | | **MN1** | | **MN2** | | **Statistics** |  |  |  |
| --- | --- | --- | --- | --- | --- | --- | --- | --- | --- | --- | --- | --- |
|  | **M** | **SD** | **M** | **SD** | **M** | **SD** | **M** | **SD** | **df1** | **df2** | **F** | **p** |
| **Duration (min.)** |  |  |  |  |  |  |  |  |  |  |  |  |
| **WASO** | 42.7 | 22.2 | 35.9 | 22.8 | 45.2 | 38.9 | 42.2 | 36.5 | 1 | 56 | 0.70 | 0.41 |
| **N1** | 39.7 | 20.2 | 36.2 | 22.2 | 48.4 | 30.9 | 37.8 | 23.1 | 1 | 55 | 1.29 | 0.26 |
| **N2** | 173.6 | 33.2 | 186.4 | 32.8 | 173.7 | 35.7 | 180.9 | 46.2 | 1 | 55 | 0.22 | 0.65 |
| **N3** | 81.1 | 34.3 | 76.7 | 32.3 | 75.8 | 35.5 | 79.6 | 42.3 | 1 | 56 | 0.09 | 0.77 |
| **R** | **75.3** | **25.8** | **81.4** | **20.6** | **69.1** | **31.2** | **74.0** | **20.0** | **1** | **55** | **4.12** | **0.05** |
|  |  |  |  |  |  |  |  |  |  |  |  |  |
| **Duration (%TST)** |  |  |  |  |  |  |  |  |  |  |  |  |
| **WASO** | 11.9 | 6.8 | 9.9 | 7.4 | 14.6 | 18.7 | 12.5 | 13.1 | 1 | 56 | 1.48 | 0.23 |
| **N1** | 10.9 | 6.0 | 9.5 | 5.9 | 13.0 | 7.9 | 10.1 | 6.2 | 1 | 55 | 1.14 | 0.29 |
| **N2** | 46.8 | 7.6 | 48.8 | 7.1 | 47.1 | 7.5 | 48.5 | 11.5 | 1 | 55 | 0.00 | 0.97 |
| **N3** | 22.0 | 9.4 | 20.3 | 8.8 | 21.4 | 11.6 | 21.6 | 11.8 | 1 | 56 | 0.13 | 0.72 |
| **R** | **20.3** | **6.7** | **21.4** | **5.2** | **18.4** | **7.6** | **19.7** | **4.5** | **1** | **55** | **4.39** | **0.04** |
|  |  |  |  |  |  |  |  |  |  |  |  |  |
| **Latency (min.)** |  |  |  |  |  |  |  |  |  |  |  |  |
| **N1** | 8.3 | 9.0 | 5.5 | 5.9 | 9.0 | 12.0 | 5.8 | 4.3 | 1 | 55 | 0.16 | 0.69* |
| **N2** | 13.5 | 9.9 | 8.7 | 5.8 | 14.1 | 13.6 | 11.4 | 8.0 | 1 | 55 | 0.93 | 0.34* |
| **N3** | 29.4 | 22.5 | 26.4 | 16.5 | 29.4 | 16.1 | 34.7 | 42.0 | 1 | 56 | 0.57 | 0.46 |
| **R** | 85.9 | 64.0 | 84.1 | 39.1 | 94.6 | 47.2 | 86.1 | 42.1 | 1 | 56 | 0.48 | 0.50 |
|  |  |  |  |  |  |  |  |  |  |  |  |  |
| **Duration (min.)** |  |  |  |  |  |  |  |  |  |  |  |  |
| **Initial N1** | 5.2 | 5.4 | 3.2 | 2.7 | 5.0 | 4.6 | 5.6 | 7.6 | 1 | 56 | 1.26 | 0.27 |
| **Initial N2** | 15.9 | 19.1 | 17.7 | 14.4 | 15.3 | 10.2 | 23.4 | 35.6 | 1 | 55 | 0.30 | 0.59 |
| **Initial N12** | 21.1 | 22.0 | 20.9 | 15.5 | 20.4 | 12.0 | 28.9 | 42.1 | 1 | 56 | 0.47 | 0.50 |

**Table S2. EEG power in several frequency bands during NREM sleep and separately during stages N2 and N3.** Delta (δ, 0.75 – 4.5 Hz), theta (θ, 4.5 – 9 Hz), alpha (α, 9 – 12 Hz), sigma (σ, 12 - 15 Hz) and beta (β, 15 – 25 Hz). Artefacts were marked during visual inspection of the data. A linear mixed model with condition, night (first or second consecutive night) and the interaction between condition and night as fixed factors and participant as random factor was used. Df1, df2, F and p of the main effect of Condition are given in the table. Bold numbers indicate significant effects of condition (p < 0.05). Star indicates significant main effect of night (p<0.05). No significant interaction effects were present.

|  | **BN1** | | **BN2** | | **MN1** | | **MN2** | | **Statistics** |  |  |  |
| --- | --- | --- | --- | --- | --- | --- | --- | --- | --- | --- | --- | --- |
|  | **M** | **SD** | **M** | **SD** | **M** | **SD** | **M** | **SD** | **df1** | **df2** | **F** | **p** |
| **NREM sleep** |  |  |  |  |  |  |  |  |  |  |  |  |
| **δ (µV²)** | **210.1** | **92.8** | **232.0** | **113.1** | **188.2** | **70.8** | **187.5** | **77.3** | **1** | **43** | **4.86** | **0.03** |
| **θ (µV²)** | 25.8 | 10.9 | 25.2 | 10.4 | 25.7 | 10.9 | 24.1 | 9.9 | 1 | 45 | 1.08 | 0.30* |
| **α (µV²)** | 15.2 | 9.2 | 15.1 | 9.3 | 15.5 | 9.7 | 14.6 | 8.7 | 1 | 45 | 0.17 | 0.68 |
| **σ (µV²)** | 8.0 | 4.7 | 8.0 | 4.7 | 8.1 | 4.9 | 7.8 | 4.5 | 1 | 45 | 0.09 | 0.76 |
| **β (µV²)** | 1.8 | 0.8 | 1.8 | 0.9 | 1.9 | 1.1 | 1.8 | 0.7 | 1 | 45 | 0.07 | 0.80 |
|  |  |  |  |  |  |  |  |  |  |  |  |  |
| **N2** |  |  |  |  |  |  |  |  |  |  |  |  |
| **δ (µV²)** | 141.8 | 46.6 | 136.7 | 51.7 | 140.5 | 47.0 | 132.1 | 43.3 | 1 | 42 | 0.15 | 0.70 |
| **θ (µV²)** | 24.4 | 9.9 | 23.8 | 9.5 | 24.3 | 9.9 | 22.9 | 9.1 | 1 | 45 | 0.70 | 0.41 |
| **α (µV²)** | 14.9 | 8.9 | 14.6 | 8.9 | 14.8 | 8.9 | 14.1 | 8.2 | 1 | 45 | 0.90 | 0.35 |
| **σ (µV²)** | 8.9 | 5.6 | 8.7 | 5.6 | 8.7 | 5.5 | 8.4 | 5.1 | 1 | 45 | 1.64 | 0.21 |
| **β (µV²)** | 2.1 | 1.1 | 2.0 | 1.0 | 2.1 | 1.2 | 1.9 | 0.9 | 1 | 45 | 0.11 | 0.74 |
|  |  |  |  |  |  |  |  |  |  |  |  |  |
| **N3** |  |  |  |  |  |  |  |  |  |  |  |  |
| **δ (µV²)** | **343.2** | **121.4** | **349.3** | **138.9** | **337.0** | **128.5** | **306.5** | **93.9** | **1** | **44** | **4.45** | **0.04** |
| **θ (µV²)** | 28.5 | 12.0 | 28.5 | 12.3 | 28.5 | 12.1 | 26.9 | 10.7 | 1 | 45 | 1.05 | 0.31 |
| **α (µV²)** | 16.1 | 10.1 | 16.1 | 10.5 | 16.9 | 12.0 | 15.5 | 10.0 | 1 | 45 | 0.02 | 0.88 |
| **σ (µV²)** | 6.4 | 3.8 | 6.3 | 3.7 | 6.8 | 4.3 | 6.5 | 3.6 | 1 | 45 | 1.99 | 0.16 |
| **β (µV²)** | 1.3 | 0.6 | 1.3 | 0.7 | 1.4 | 1.0 | 1.4 | 0.6 | 1 | 45 | 1.91 | 0.17 |

**Table S3. Slow waves occurring during all NREM sleep and during stages N2 and N3.** A linear mixed model with condition, night (first or second consecutive night) and the interaction between condition and night as fixed factors and participant as random factor was used. Df1, df2, F and p of the main effect of Condition are given in the table. Bold numbers indicate significant effects of condition (p < 0.05). Star indicates significant main effect of night (p < 0.05). No significant interaction effects were present.

|  | **BN1** | | **BN2** | | **MN1** | | **MN2** | | **Statistics** |  | |  |  |
| --- | --- | --- | --- | --- | --- | --- | --- | --- | --- | --- | --- | --- | --- |
|  | **M** | **SD** | **M** | **SD** | **M** | **SD** | **M** | **SD** | **df1** | | **df2** | **F** | **p** |
| **NREM sleep** |  |  |  |  |  |  |  |  |  | |  |  |  |
| n | 1254.0 | 868.7 | 1145.2 | 720.6 | 1123.1 | 913.7 | 1038.8 | 574.5 | 1 | | 46 | 1.77 | 0.19 |
| Density (#/20s) | 1.7 | 0.6 | 1.6 | 0.6 | 1.5 | 0.6 | 1.5 | 0.5 | 1 | | 46 | 2.11 | 0.15 |
| Frequency (Hz) | 0.7 | 0.0 | 0.7 | 0.0 | 0.7 | 0.0 | 0.7 | 0.1 | 1 | | 46 | 0.00 | 0.99 |
| Duration (s) | 1.4 | 0.1 | 1.4 | 0.1 | 1.4 | 0.0 | 1.5 | 0.1 | 1 | | 46 | 0.00 | 0.95 |
| Amplitude (µV) | -0.7 | 4.3 | -0.6 | 4.7 | -1.4 | 4.7 | -0.4 | 3.9 | 1 | | 46 | 0.12 | 0.73 |
|  |  |  |  |  |  |  |  |  |  | |  |  |  |
| **N2** |  |  |  |  |  |  |  |  |  | |  |  |  |
| n | 324.9 | 205.9 | 296.1 | 185.6 | 274.6 | 110.6 | 343.3 | 242.5 | 1 | | 46 | 0.00 | 0.96 |
| Density (/20s) | 1.1 | 0.4 | 1.0 | 0.3 | 0.9 | 0.1 | 1.0 | 0.4 | 1 | | 46 | 0.46 | 0.50 |
| Frequency (Hz) | 0.7 | 0.0 | 0.7 | 0.0 | 0.7 | 0.0 | 0.7 | 0.1 | 1 | | 46 | 0.01 | 0.93 |
| Duration (s) | 1.4 | 0.1 | 1.4 | 0.1 | 1.4 | 0.1 | 1.4 | 0.2 | 1 | | 46 | 0.00 | 0.96 |
| Ampl. (µV) | 0.6 | 5.3 | 0.1 | 6.2 | -0.4 | 6.5 | 0.7 | 5.6 | 1 | | 46 | 0.05 | 0.83 |
|  |  |  |  |  |  |  |  |  |  | |  |  |  |
| **N3** |  |  |  |  |  |  |  |  |  | |  |  |  |
| n | 929.1 | 787.9 | 849.1 | 627.6 | 848.5 | 823.8 | 695.6 | 573.3 | 1 | | 46 | 3.03 | 0.09 |
| Density (/20s) | 2.1 | 0.9 | 2.0 | 0.8 | 2.0 | 0.8 | 1.8 | 0.7 | 1 | | 46 | 3.48 | 0.07 |
| Frequency (Hz) | **0.73** | **0.03** | **0.73** | **0.03** | **0.74** | **0.02** | **0.74** | **0.03** | **1** | | **46** | **7.01** | **0.01** |
| Duration (s) | **1.43** | **0.07** | **1.44** | **0.07** | **1.42** | **0.05** | **1.41** | **0.07** | **1** | | **46** | **6.39** | **0.01** |
| Ampl. (µV) | -1.3 | 4.4 | -0.6 | 4.6 | -1.9 | 4.6 | -0.7 | 3.1 | 1 | | 46 | 0.17 | 0.68 |

**Figure S1. Memory task protocol.** Declarative memory performance was assessed using a previously-described word-pair task (German word pairs) (Plihal & Born, 1997).

**
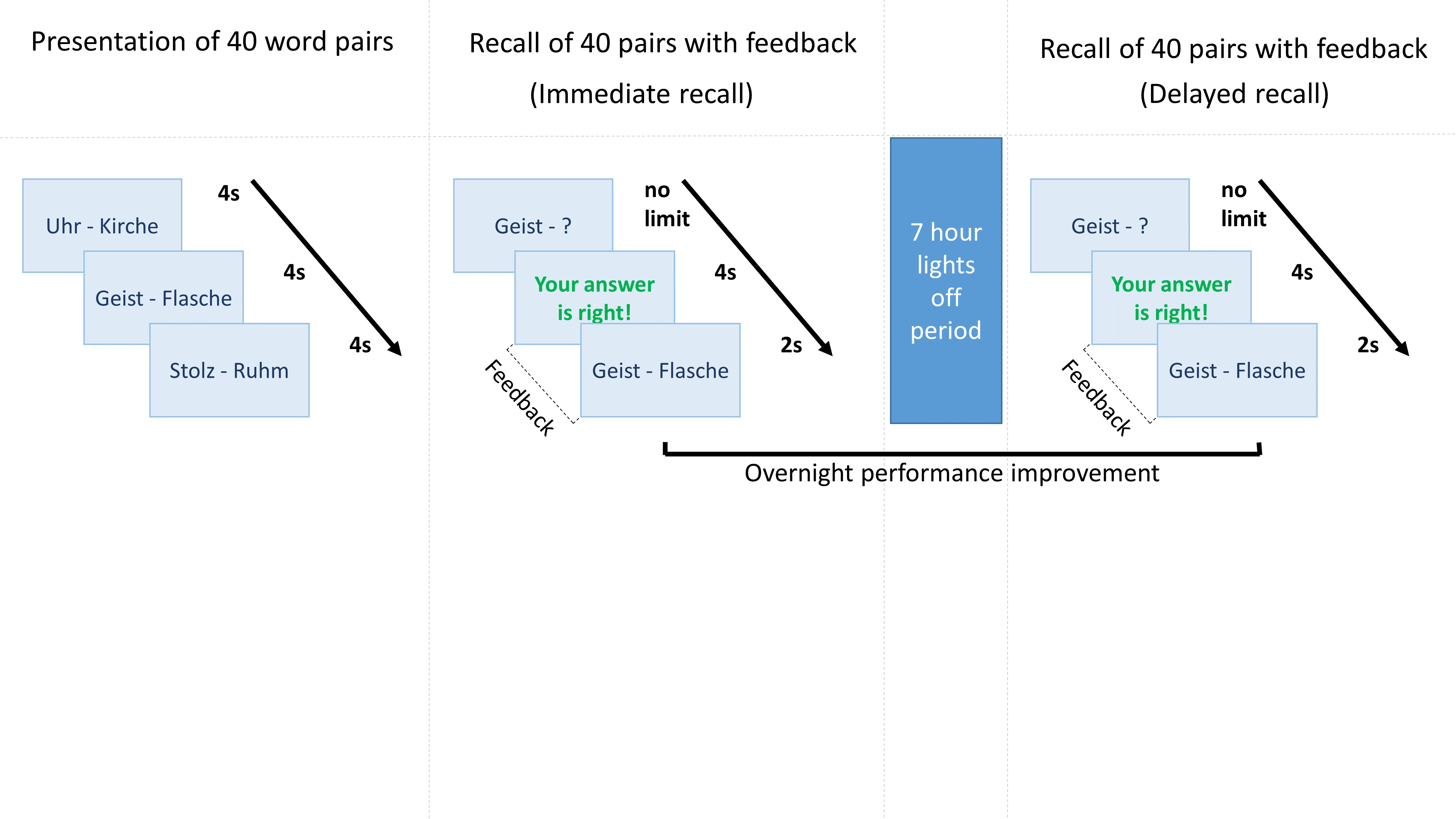
**

**Table S4. Sleep spindles and memory-task performance.** Spindles with a frequency between 12 – 15 Hz and a duration between 0.5 and 3 s were automatically detected. The average frequency, duration, maximum amplitude, integrated absolute amplitude and activity (integrated absolute amplitude/duration) of each spindle were determined. Declarative memory performance was assessed using a word-pair task. Immediate recall (IR) took place directly after learning the word pairs 1 hour before lights off. Delayed recall (DR) took place 30 min after lights on. In the memory task two data points are missing, one during baseline night 2 and one in movement night 1. A linear mixed model with condition, night (first or second consecutive night) and the interaction between condition and night as fixed factor and participant as random factor was used. Df1, df2, F and p of the main effect of Condition are given in the table. Bold numbers indicate significant effects of condition (p < 0.05). Star indicates significant main effect of night (p < 0.05). Two stars indicate a significant main effect of night, as well as an interaction effect between condition and night (p < 0.05) were present.

|  | **BN1** | | **BN2** | | **MN1** | | **MN2** | | **Statistics** | |  |  |
| --- | --- | --- | --- | --- | --- | --- | --- | --- | --- | --- | --- | --- |
|  | **M** | **SD** | **M** | **SD** | **M** | **SD** | **M** | **SD** | **df1** | **df2** | **F** | **p** |
| **Spindles (12-15Hz)** | n=19 |  |  |  |  |  |  |  |  |  |  |  |
| Count (#) | 1.5 | 0.7 | 1.7 | 0.7 | 1.5 | 0.6 | 1.6 | 0.6 | 1 | 54 | 0.53 | 0.47 |
| Density (#/20s) | 375.8 | 201.8 | 424.1 | 187.8 | 369.7 | 175.0 | 397.7 | 163.3 | 1 | 54 | 0.62 | 0.44 |
| Duration (s) | 1.1 | 0.2 | 1.1 | 0.1 | 1.0 | 0.1 | 1.0 | 0.1 | 1 | 54 | 0.97 | 0.33 |
| Peak amplitude (µV) | 17.3 | 12.9 | 16.0 | 11.7 | 13.6 | 3.8 | 13.2 | 3.6 | 1 | 54 | 2.91 | 0.09 |
| Mean frequency (Hz) | 13.7 | 0.3 | 13.7 | 0.3 | 13.7 | 0.3 | 13.7 | 0.3 | 1 | 54 | 0.01 | 0.92 |
| Integrated amplitude (µV) | 849.5 | 618.8 | 752.3 | 427.0 | 660.0 | 221.0 | 649.6 | 240.6 | 1 | 54 | 3.86 | 0.05 |
| Integrated activity (µV/min) | 6.2 | 4.4 | 5.5 | 3.0 | 4.9 | 1.4 | 4.8 | 1.3 | 1 | 54 | 2.99 | 0.09 |
|  |  |  |  |  |  |  |  |  |  |  |  |  |
| **Memory Task** | n=19 | | |  |  |  |  |  |  |  |  |  |
| Immediate recall (IR) | 14.6 | 6.9 | 15.8 | 7.0 | 11.4 | 6.4 | 16.1 | 7.5 | 1 | 52 | 1.88 | 0.18* |
| Delayed recall (DR) | 17.9 | 7.7 | 17.2 | 7.8 | 14.2 | 6.5 | 19.0 | 9.0 | 1 | 52 | 1.05 | 0.31** |
| Overnight performance improvement (DR-IR) | 3.3 | 3.5 | 1.5 | 2.5 | 2.8 | 4.1 | 2.9 | 3.7 | 1 | 53 | 0.58 | 0.45 |
| Initial acquisition rate (IR/DR*100) | 80.4 | 17.1 | 100.0 | 40.7 | 88.1 | 47.2 | 90.8 | 32.4 | 1 | 53 | 0.01 | 0.92 |

**References**

Iber, C., Ancoli-Israel, S., Chesson, A., & Quan, S. (2007). *The AASM manual for the scoring of sleep and associated events: rules, terminology, and technical specification* (1st ed.). Westchester, IL: American Academy of Sleep Medicine.

Plihal, W., & Born, J. (1997). Effects of early and late nocturnal sleep on declarative and procedural memory. *Journal of cognitive neuroscience, 9*(4), 534-547.
